# Extraction and Characterization of Hemicelluloses from a Softwood Acid Sulfite Pulp

**DOI:** 10.3390/polym13132044

**Published:** 2021-06-22

**Authors:** Pauline Vincent, Frédérique Ham-Pichavant, Christelle Michaud, Gérard Mignani, Sergio Mastroianni, Henri Cramail, Stéphane Grelier

**Affiliations:** 1 CNRS, University Bordeaux, Bordeaux INP, LCPO, UMR 5629, 33600 Pessac, France; vincent-pauline@live.fr (P.V.); frederique.pichavant@enscbp.fr (F.H.-P.); 2 Rayonier AM France Innovation, 33174 Gradignan, France; Christelle.Michaud@rayonieram.com; 3 Research and Innovation Center of Lyon, Solvay, 85 Avenue des Frères Perret, 69192 Saint Fons, France; gerard.mignani@solvay.com (G.M.); sergio.mastroianni@solvay.com (S.M.)

**Keywords:** softwood, sulfite pulp, hemicelluloses extraction, glucomannans, methylglucuronoxylans

## Abstract

Hemicelluloses were extracted from a softwood acid sulfite pulp in a three-step procedure. Further delignification step resulted in a holocellulose pulp containing only 1.7 wt.% of the lignin left. Cold caustic extraction (CCE) with 18 wt.% NaOH at 60 °C for 1 h was performed to solubilize hemicelluloses of the holocellulose. An unbleached cellulose pulp was then obtained 97% pure, which indicates that 89% of the hemicelluloses were removed. After purification, extraction yields between 1.1 wt.% and 9.5 wt.% were obtained from the delignified pulp and the hemicelluloses’ chemical compositions and structures were investigated by ^1^H, ^13^C nuclear magnetic resonance spectroscopy (NMR) and two-dimensional NMR by correlation spectroscopy (2D-COSY) and proton-detected heteronuclear single-quantum correlation (2D-HSQC), high-performance anion-exchange chromatography coupled with a pulsed amperometry detector (HPAEC-PAD), size-exclusion chromatography coupled with a refractive index detector (SEC-RI) and thermogravimetric analyses (TGA). Hemicelluloses were obtained with a purity of 96%, with short cellulosic chains as the only residue. Sulfite pulping modified the hemicelluloses’ structure, and it was found that two types of hemicelluloses were isolated, glucomannans, predominant at 67%, and methylglucuronoxylans. Finally, alkali-soluble hemicelluloses displayed relatively narrow size distributions and low molar masses, M_w_ varying between 18,900 and 30,000 g/mol after acid sulfite pulping.

## 1. Introduction

Sustainable development, climate change and environmental health issues are today the subjects of big concern [1]. There is, in particular, a strong need in obtaining traditional oil-based chemicals from biobased feedstock. Biorefineries are therefore exploring renewable sources for the production of energy and chemicals. Among the renewable resources, lignocellulosic biomass is the most abundant on the Earth and offers a significant amount of carbon raw materials, such as cellulose, hemicelluloses, lignin and many other derivatives [2].

Cellulose-based products are mainly obtained from wood and plant chemical processes (70% of the production) which are pH-dependent with basic kraft and acid sulfite pulping [3]. Cellulosic pulps are mostly intended for the paper industry, but also for dissolving pulp industries, including viscose, cellulose acetate, cellulose nitrate or cellulose ether applications. Those cellulose-derived products require high purity of the cellulose by removing the remaining lignin and hemicellulose side products [4].

Hemicelluloses are short-branched heteropolysaccharides and represent 25–40% of the wood biopolymers [2,4,5]. Xylans are the most abundant hemicelluloses in hardwoods (15–30%), glucomannans—in softwoods (10–25%) [6]. In acidic conditions such as in sulfite pulping, hemicelluloses are hydrolyzed and reduced to their monosaccharide units. In basic conditions, hemicelluloses are either depolymerized to form aliphatic carboxylic acids [7] or solubilized [8]. In both cases, coproducts are dissolved in cooking or extraction liquors which are combusted to produce energy, but organic chemicals are then not fully valorized [9]. As already proposed in other industries such as food processing, the utilization of byproducts increases the sustainability and reduces waste [10]. These hemicelluloses from the sulfite pulp could provide valuable raw materials for use in food, cosmetic and pharmaceutical industries. They also have a great potential as biobased polymers [11].

Hemicellulose extraction directly from the biomass can be achieved in three or four different steps depending on the biomass origin, wood or plants, and starting from powder or pellets [12,13,14,15,16]. The second step of delignification, either with sodium chlorite solutions [13,17,18] or hydrogen peroxide [19,20], is used to obtain a holocellulose material. Two techniques are used to extract hemicelluloses from the holocellulose: organosolv treatment [21,22] and, more generally, alkaline extractions [13,17,18,23]. Hemicellulose extraction from the cellulosic pulp has also already been reported in the literature using acidic and caustic extraction methods [24], hot water extraction [25] and enzymatic treatments [26,27].

Herein, we report the extraction of hemicelluloses from an unbleached cellulosic sulfite pulp in three steps to obtain pure hemicelluloses from a cellulosic pulp which can be entirely valorized. Indeed, all the components of wood are recovered: lignosulfonate—after sulfite cooking, hemicelluloses—with this process, unbleached cellulose (with high purity)—obtained at the same time. Sodium chlorite has been chosen to perform the delignification step, followed by cold caustic extraction from the holocellulose to solubilize hemicelluloses by cellulose swelling and the last step of hemicellulose recovery and purification. The hemicelluloses’ structures were analyzed by 1D and 2D nuclear magnetic resonance spectroscopy (NMR), the hemicelluloses’ proportions—by high-performance anion-exchange chromatography (HPAEC-PAD), size-exclusion chromatography (SEC) and thermogravimetric analyses (TGA).

## 2. Experiment

### 2.1. Materials

The cellulosic pulp provided by Rayonier Advanced Materials (RYAM, Tartas, France) is produced using the acid sulfite pulping process from softwood maritime pine. Reagents, such as sodium chlorite (80%), sodium hydroxide (98%), sodium acetate (99%) and hydrochloric acid (37%) were obtained from Fisher Scientific (Illkirch, France). All the reagents were used without further purification. Absolute ethanol was purchased from VWR, and standard compounds used for ion chromatography calibration, including glucose, mannose, xylose, arabinose and galactose were purchased from Sigma-Aldrich (St. Quentin Fallavier, France).

### 2.2. Hemicellulose Extraction Procedure

**Figure 1:**
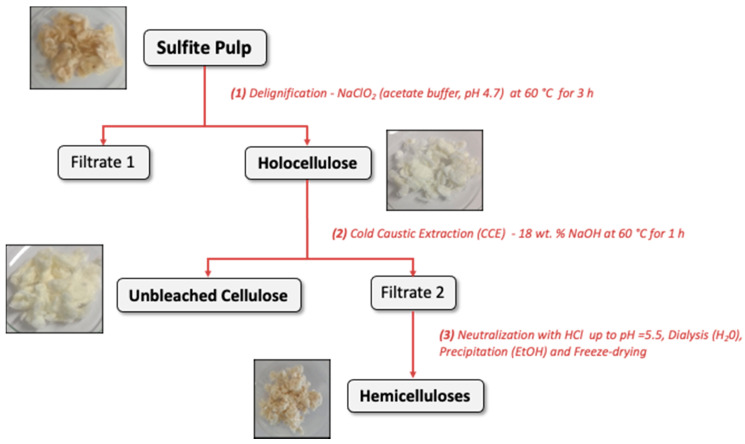
Procedure for hemicellulose extraction from a sulfite pulp.

The three-step procedure of hemicellulose extraction from a sulfite cellulosic pulp is illustrated in Figure 1.

Step 1: delignification. The pulp was immersed with a dry solid to liquid ratio of 1/60 (g/mL) in an acetate buffer solution at pH 4.7 (10 g acetic acid and 13 g sodium acetate per liter) and disintegrated with a rod stirrer for 3 min. Two thirds of the initial volume of a solution of sodium chlorite in the acetate buffer at 34 wt.% were added. The mixture was heated at 60 °C and magnetically stirred at 500 rpm for 3 h. After the treatment, the delignified product named holocellulose was filtered off, washed with distilled water and further dried in an oven at 40 °C for 24 h. The solid content was determined to obtain the extraction yield of holocellulose.

Step 2: cold caustic extraction (CCE). The holocellulose was extracted using an 18 wt.% NaOH solution with a dry solid to liquid ratio of 1/25 (g/mL). The mixture was magnetically stirred at 500 rpm and 60 °C for 1 h. The residue of crude cellulose was filtered off and washed with distilled water and further dried in an oven at 40 °C for 24 h. The solid content was determined to obtain the extraction yield of crude cellulose.

Step 3: hemicellulose purification. The filtrate was obtained after CCE was acidified with HCl at 6 M until pH reached 5.5. After concentration under reduced pressure, dialysis against water using regenerated cellulose membranes (cutoff, 1 kDa) was set up for three days to remove salts. Hemicelluloses were precipitated with three volumes of ethanol, recovered by filtration and freeze-dried.

### 2.3. Extraction Yields

The procedure of hemicellulose (HC) extraction was repeated seven times, noted from A to F and then RYAM. For the extractions HC-A–C, the procedure started with 10 g sulfite pulp; with 20 g—for the extractions HC-D–F; with 200 g—for the HC-RYAM extraction (on the dry matter basis). The extraction yields were calculated after ash content determination by thermogravimetric analyses (TGA) of hemicellulose products and compared to the holocellulose to avoid lignocellulosic biomass origin (plants or wood) and the presence of lignin (the details are presented in Table S1 in the Supporting Information):

$$Y\;\left(\%\right)=\frac{m_{hemicelluloses}}{m_{holocellulose}}\times 100.$$

### 2.4. Pulp Characterizations

The pulps were characterized according to the standard methods: dry matter content—following ISO 628:2008; Kappa number—following ISO 302:2015; hemicellulose content, or alkali solubility at 18%, S_18_—following ISO 692:1982 and ISO 699:2015; neutral sugars and hemicellulose proportions were determined by high-performance anion-exchange chromatography with pulse amperometric detection (ISO/CD 21437) after hydrolysis following TAPPI method T 249 cm-00. The lignin content was calculated from the Kappa number with an empirical factor of 0.147 for softwood pulping.

### 2.5. Instruments Methods

Nuclear magnetic resonance (NMR). Analyses were performed at 298 K on a AVANCE 400 spectrometer Bruker (Billerica, MA, USA) operating at 400.2 MHz for ^1^H. The material in the amount of 20 mg was dissolved in 500 µL deuterium oxide, D_2_O (99.9% atom D, Eurisotop); 128 scans were recorded for ^1^H experiments, 4096—for ^13^C experiments. The two-dimensional spectra obtained by correlation spectroscopy (COSY) and proton-detected heteronuclear single-quantum correlation (HSQC) were acquired with 16 and 32 datapoints. Data processing was performed using the standard Bruker TOPSPIN software.

Size-exclusion chromatography (SEC). SEC analyses were performed on a Thermo Scientific (Waltham, MA, USA) apparatus equipped with an SB G guard column (40 × 6 mm) and two SHODEX (Tokyo, Japan) OH Pack SB 804 columns (300 × 8 mm) conditioned at 26 °C, along with a refractive index detector (model RI Optilab T-rex). The system was calibrated with dextran standards. A phosphate eluent (0.2 M NaNO_3_ and 0.01 M Na_2_HPO_4_ adjusted to pH 9) was used at the flow rate of 0.5 mL/min and the samples were dissolved at 10 mg/mL. Molar masses were determined using Chromeleon and Astra and calibrated based on the ethylene glycol elution time.

Thermogravimetric analyses (TGA). TGA analyses were performed on a Q500 system TA Instruments (New Castle, DE, USA) using a platinum pan. The samples were heated up from room temperature to 800 °C at a heating rate of 10 °C/min under nitrogen atmosphere and then under air atmosphere up to 900 °C.

Ion chromatography or high-performance anion-exchange chromatography (HPAEC-PAD) was performed on an ICS 3000 apparatus (Dionex, Sunnyvale, CA, USA) equipped with a Dionex CarboPac SA10 guard (4 × 50 mm) and a Diones CarboPac SA10 column (4 × 250 mm) conditioned at 20 °C. Detection was achieved via pulsed amperometry. A Dionex AS-DV autosampler handled volumes of injection samples of 10 µL for elution at 1 mL/min for 30 min. Three eluents were used, UHPLC grade water (eluent A), 0.01 M NaOH (eluent B) and 0.1 M NaOH (eluent C), under the conditions presented in Table 1.The chromatograms were analyzed using the Chromeleon software and monosaccharide quantification was achieved using five standards (glucose, mannose, xylose, arabinose, galactose).

From the monosaccharide quantification, hexosans (glucans, mannans, galactans) and pentosanes (xylans, arabinans) compositions are obtained according to the following ratios: $\frac{M_{anhydrohexoses}\;\left(g/mol\right)}{M_{hexoses\;}\left(g/mol\right)}=\frac{162}{180}=0.9$ and $\frac{M_{anhydropentoses}\;\left(g/mol\right)}{M_{pentoses}\left(g/mol\right)}=\frac{132}{150}=0.88$. Finally, the cellulose is obtained as follows: $cellulose\;\left(\%\right)=\frac{m_{glucans}-m_{mannans}-m_{galactans}}{m_{sample}}\times 100$.

## 3. Results and Discussion

### 3.1. Initial Pulp Characterizations

The initial pulp provided by RYAM is obtained by the acid sulfite pulping process from softwood maritime pine. Hemicelluloses, lignin and dry matter content of the pulp were determined as presented in Table 2**.** The initial biopolymer composition of the pulp indicates a delignification degree of 74% and hemicellulose hydrolysis between 38% and 4% during acid sulfite pulping of maritime pine. However, the hemicellulose content still represents 15.4 wt.% of the total dry matter.

### 3.2. Pulp Characterizations at Different Extraction Steps

**Figure 2:**
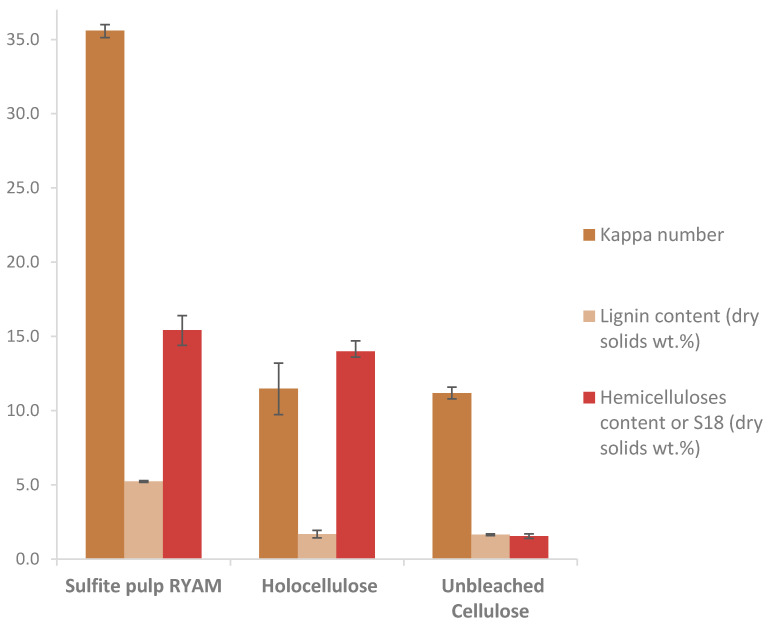
Evolution of the lignin and hemicellulose contents in pulps during extraction.

The efficiency of the procedure was evaluated by determining the lignin and hemicellulose content in each pulp. The results are presented in Figure 2.

For the delignification step leading to the holocellulose, 1.7 wt.% lignin was left compared to 5.2 wt.% in the initial sulfite pulp. In the end, almost 92% of the lignin in maritime pine was removed after sulfite cooking, and the additional sodium chlorite step removed 67% of the lignin. A slight decrease in S_18_ values, from 15.4 ± 1.0% to 14.0 ± 0.5% (Figure 2), shows that hemicelluloses were also hydrolyzed during the first step (almost 9%). Lignin chemically linked to hemicelluloses, also called lignin–carbohydrate complexes (LCC), could explain this loss.

During cold caustic extraction of the holocellulose, there was no variation of the kappa number in the unbleached cellulose which indicates that only polysaccharides were extracted in this step. A decrease of S_18_, from 14.0 ± 0.5% to 1.65 ± 0.15%, proves that 89% of hemicelluloses were solubilized during this second step. In the end, the unbleached cellulose pulp obtained with this procedure had a purity of 96.8%.

### 3.3. Extraction Yields

**Figure 3:**
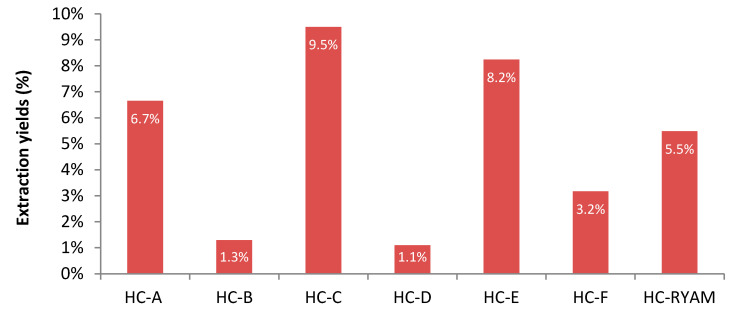
Extraction yields of hemicelluloses from the sulfite pulp as compared to the holocellulose.

As shown in Figure 3, there is a large disparity in hemicellulose yields, from 1.1 wt.% to 9.5 wt.%, and for the same initial weight. Pulp inhomogeneity and macromolecule repartition in the pulp could be responsible for such extraction yields. An average extraction yield of 5.1 wt.% was obtained against 14.0 wt.% in the holocellulose, which indicates that 36 wt.% of hemicelluloses were extracted from a delignified material. There are still limiting factors like hemicellulose degradation during each treatment or incomplete hemicellulose precipitation using ethanol. Yields between 1.8% and 10.5% were obtained in three steps directly from wood after sodium chlorite delignification, caustic extraction and ethanol precipitation [13,18].

### 3.4. Hemicellulose Characterizations

In softwoods, native hemicelluloses are galactoglucomannans (10–25%), arabinoglucuronoxylans (5–10%) and arabinogalactans (5–35%) [6]. Most likely, conditions used in pulping treatment change lignin and hemicellulose structures. 

#### 3.4.1. Structural Identification

**Figure 4:**
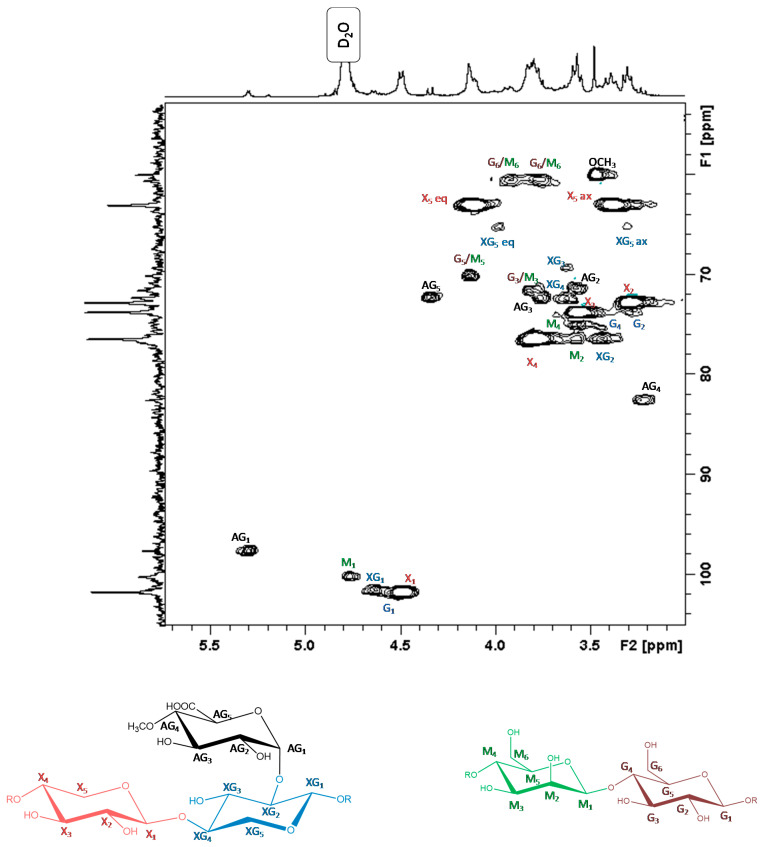
HSQC spectrum of hemicelluloses in D_2_O. The following designations are used: G—glucose units, M—mannose units, X—non-substituted xylose units, XG—glucuronic acid substituted xylose units, AG—glucuronic acid units. The numbers refer to the H and C atoms.

Examination of the HSQC NMR spectrum (see Figure 4), revealed five different anomeric groups at 5.30 and 4.50 ppm (97.59 and 101.74 ppm in ^13^C, respectively). Among those, four signals were assigned to β-units (reported between 4.5 and 5 ppm in ^1^H NMR), one signal—to α-units (reported between 5 and 5.3 ppm) [28]. Two-dimensional proton–proton spectroscopy showed correlation between the adjacent protons in ^3^J up to five or six protons, respectively, for pentose or hexose units. Three pentose units and two hexose units were identified, and the signals were attributed to β-xylose, glucuronic acid substituted β-xylose, β-mannose, β-glucose and α-glucuronic acid [28,29,30]. No signals were attributed to arabinose or galactose units. Acidolysis of α-arabinose units in common xylans and α-galactose units of galactoglucomannans in softwoods are reported in the literature [13,31]. The galactoglucomannans are readily depolymerized by acids, especially the ether bond between the side group galactose and the main chain. In addition, because of their furanosidic structure, the arabinose side chains are also readily hydrolyzed by acids (6). Total assignation of hemicelluloses, ^1^H NMR and ^13^C NMR are presented in the Supporting Information (Figures S1 and S2 and Table S2).

Finally, two types of hemicelluloses were identified: glucomannans [32,33] and methylglucuronoxylans [18,34,35]. In fact, the acidic function of α-glucuronic acid was assigned at 176.8 ppm in ^13^C NMR, the methoxy group—at 3.48 ppm in ^1^H NMR and 59.98 ppm in ^13^C NMR.

#### 3.4.2. Hemicellulose Proportions

Since there was no variation of the lignin content between the holocellulose and unbleached cellulose (1.69 to 1.65 wt.%), hemicelluloses were considered pure. HPAEC-PAD was used to determine proportions between isolated glucomannans and methylglucuronoxylans. Table 3 presents mono- and polysaccharide quantification obtained by conventional ion chromatography. Only 4 wt.% of glucans, such as cellulose chains, were found in the hemicellulose fractions. The low concentration of arabinose and galactose sugars confirmed NMR interpretations and the absence of branched arabinose and galactose units or arabinogalactans which can be found in native softwoods due to their fast hydrolysis in the acidic environment provided by sulfite pulping. The proportion of glucomannans was also found higher than of methylglucuronoxylans (67% compared to 33%) as in softwoods, with an average mannose/glucose ratio of 9:2.

#### 3.4.3. Hemicellulose Molecular Weights

Molar masses of the extracted hemicelluloses were obtained by SEC-RI showing bimodal distributions for each extraction analysis (see Figure S3). The first population eluted at 23 min could correspond either to aggregates of branched polysaccharides or longer cellulose chains, whereas elutions of the extracted hemicelluloses were attributed at 28 min. The average molar masses values M_W_, M_n_ and dispersity Ɖ of the isolated hemicelluloses are given in Table 4. The hemicelluloses displayed M_W_ values varying between 18,900 and 30,000 g/mol, with a dispersity of 1.4 to 2.0. In the literature, molar masses between 32,790 and 78,660 g/mol are reported for the hemicelluloses isolated in a three-step procedure directly from wood [13,18].

## 4. Conclusions

Two types of hemicelluloses, glucomannans and methylglucuronoxylans, were isolated from a softwood cellulosic pulp after sulfite pulping. The modified three-step procedure commonly used on wood materials consists in sodium chlorite delignification, cold caustic extraction using 18 wt.% NaOH and purification by ethanol precipitation. This method was found repeatable in terms of chemical structure and composition of hemicelluloses, but extraction yields between 1.1 wt.% and 9.5 wt.% were obtained. Compared to native softwood hemicelluloses, no traces of arabinose or galactans were found due to the acidic pulping conditions.

Unbleached cellulose was then obtained with a purity of 97%, which could be used in specialty cellulose applications, and alkali-soluble hemicelluloses could be used directly without further acidic hydrolysis or basic depolymerization towards the design of original functional biomaterials. Moreover, these hemicelluloses could be further used as raw materials to investigate the formation of hydroxy acids such as lactic or glycolic acids during CCE after the treatment of unbleached pulp.

## Figures and Tables

**Table 1:** Eluent conditions of HPAEC-PAD analysis.

Time (Min)	Eluent A (%)	Eluent B (%)	Eluent C (%)
0	90	10	0
8	90	10	0
8.1	0	0	100
15	0	0	100
15.1	90	10	0
30	90	10	0

**Table 2:** Biopolymer composition of common softwood and sulfite pulps [2,10] compared to the RYAM sulfite pulp.

	Maritime Pine	Sulfite Pulps	Sulfite Pulp, RYAM *
Dry matter	-	-	29.6 ± 0.2 (%)
Cellulose	42–50%	83–87%	79.7 ± 0.6 (%)
Hemicelluloses (S18)	24–27%	10–15%	15.4 ± 1.0 (%)
Lignin (Kappa *, 0.147)	20%	2–5%	5.2 ± 0.1 (%)

* Each analysis was repeated three times.

**Table 3:** Mono- and polysaccharide proportions in the hemicelluloses extracted from a sulfite pulp by HPAEC-PAD.

	Proportions (wt.%)
**Monosaccharides**	100
Arabinose	0.1
Galactose	0.4
Glucose	16.5
Mannose	51.0
Xylose	32.0
**Hemicelluloses**	96
Glucomannans	64
Methylglucuronoxylans	32
**Cellulose**	4

**Table 4:** Weight-average (Mw) and number-average (M_n_) molar masses and dispersity (Ɖ = M_w_/M_n_) of the hemicelluloses extracted from a sulfite pulp.

	M_W_ (g/mol)	M_n_ (g/mol)	Ɖ
**HC-A**	26,400	15,700	1.7
**HC-B**	18,900	13,000	1.5
**HC-C**	30,000	17,600	1.7
**HC-D**	19,800	14,000	1.4
**HC-E**	26,600	13,800	1.9
**HC-F**	25,000	12,400	2.0
**HC-RYAM**	24,000	13,500	1.8

## Data Availability

Data sharing not applicable.

## Supplementary Materials

The following are available online at https://www.mdpi.com/article/10.3390/polym13132044/s1, Figure S1: ^1^H NMR spectrum of hemicelluloses extracted from the sulphite pulp in D_2_O. (glucose units in brown, man-nose units in green, non-substituted xylose units in red, glucuronic acid substituted xylose units in blue, glucuronic acid units in black. The number refers to the H-atom; Figure S2: ^13^C NMR spectrum of hemicelluloses extracted from the sulphite pulp in D_2_O. (glucose units in brown, mannose units in green, non-substituted xylose units in red, glucuronic acid substituted xylose units in blue, glucuronic acid units in black. The number refers to the C-atom; Figure S3: SEC-RI chromatograms of hemicelluloses extracted from the sulfite pulp; Table S1: Results and yields obtained during hemicelluloses extraction procedure; Table S2: Total signals assignation of hemicelluloses extracted from the sulfite pulp in D_2_O.
