# Supplementary material for: Extraction and Characterization of Hemicelluloses from a Softwood Acid Sulfite Pulp
Source: Polymers (Basel). 2021 Jun 22;13(13):2044. doi: 10.3390/polym13132044 (PMC8271795; doi:10.3390/polym13132044)
Supplement: Supplementary file 1 [file polymers-13-02044-s001.zip › polymers-1254542-supplementary.pdf]

Supporting Information

Extraction and Characterization of Hemicelluloses from a Softwood Acid Sulfite Pulp

Pauline Vincent [1], Frédérique Ham-Pichavant [1], Christelle Michaud [2], Gérard Mignani [3], Sergio Mastroianni [3], Henri Cramail [1,*] and Stéphane Grelier [1,*]

[1] CNRS, University Bordeaux, Bordeaux INP, LCPO, UMR 5629, F-33600 Pessac, France; vincent-pauline@live.fr (P.V.); frederique.pichavant@enscbp.fr (F.H.-P.)
[2] Rayonier AM France Innovation, 33174 Gradignan, France; Christelle.Michaud@rayonieram.com
[3] Research and Innovation Center of Lyon, Solvay, 85 Avenue des Frères Perret, 69192 Saint Fons, France; gerard.mignani@solvay.com (G.M.); sergio.mastroianni@solvay.com (S.M.)
* Correspondence: henri.cramail@enscbp.fr (H.C.); stephane.grelier@enscbp.fr (S.G.)

Citation: Vincent, P.; Ham-Pichavant, F.; Michaud, C.; Mignani, G.; Mastroianni, S.; Cramail, H.; Grelier, S. Extraction and Characterization of Hemicelluloses from a Softwood Acid Sulfite Pulp. *Polymers* **2021**, *13*, x. https://doi.org/10.3390/xxxxx

Academic Editor:Jean Duhamel

Received: 25 May 2021
Accepted: 17 June 2021
Published: date

Publisher's Note: MDPI stays neutral with regard to jurisdictional claims in published maps and institutional affiliations.

Copyright: © 2021 by the authors. Licensee MDPI, Basel, Switzerland. This article is an open access article distributed under the terms and conditions of the Creative Commons Attribution (CC BY) license (http://creativecommons.org/licenses/by/4.0/).

Table S1. Results and yields obtained during hemicelluloses extraction procedure.

	m initial sulfite pulp (g)	m holocellulose (g)	Delignification Yields (%)	m initial Holocellulose (g)	m CUnbleached Cellulose (g)	CCE Yields (%)	m hemicelluloses (g)	Hemicelluloses Yields (%)
**HC-A**	10.00	9.62	96.2	9.62	8.85	92.0	0.64	6.7
**HC-B**	10.00	8.52	85.2	8.52	7.33	86.0	0.11	1.3
**HC-C**	9.87	8.69	88.0	4	2.68	67.0	0.38	9.5
**HC-D**	19.77	18.19	92.0	18.19	16.37	90.0	0.2	1.1
**HC-E**	20.00	16.02	80.1	16.02	12.50	78.0	1.32	8.2
**HC-F**	19.99	18.43	92.2	15.13	13.42	88.7	0.48	3.2
**HC-RYAM**	196.96	141.81	72.0	135.74	102.23	51.9	7.44	5.5

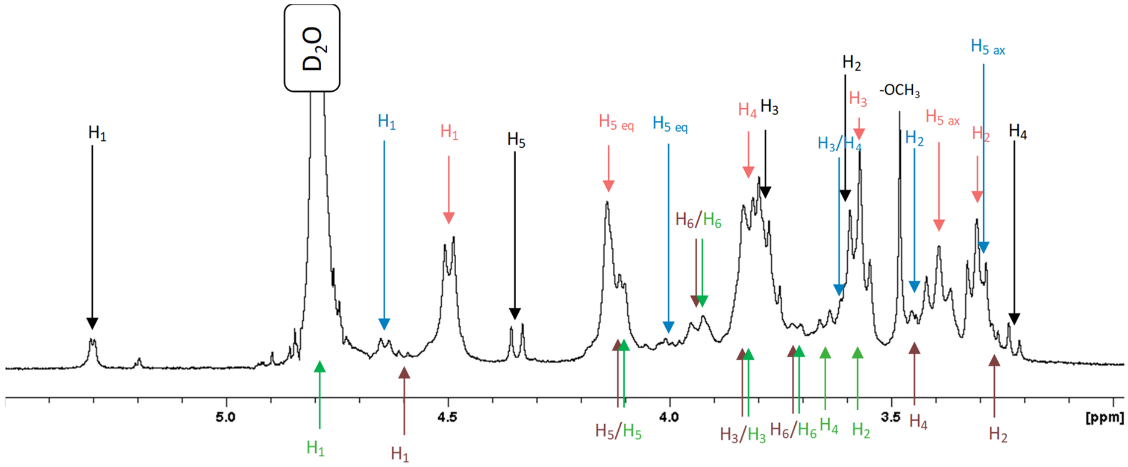

Figure S1. ^{1}H NMR spectrum of hemicelluloses extracted from the sulphite pulp in D_2O. (glucose units in brown, mannose units in green, non-substituted xylose units in red, glucuronic acid substituted xylose units in blue, glucuronic acid units in black. The number refers to the H-atom.

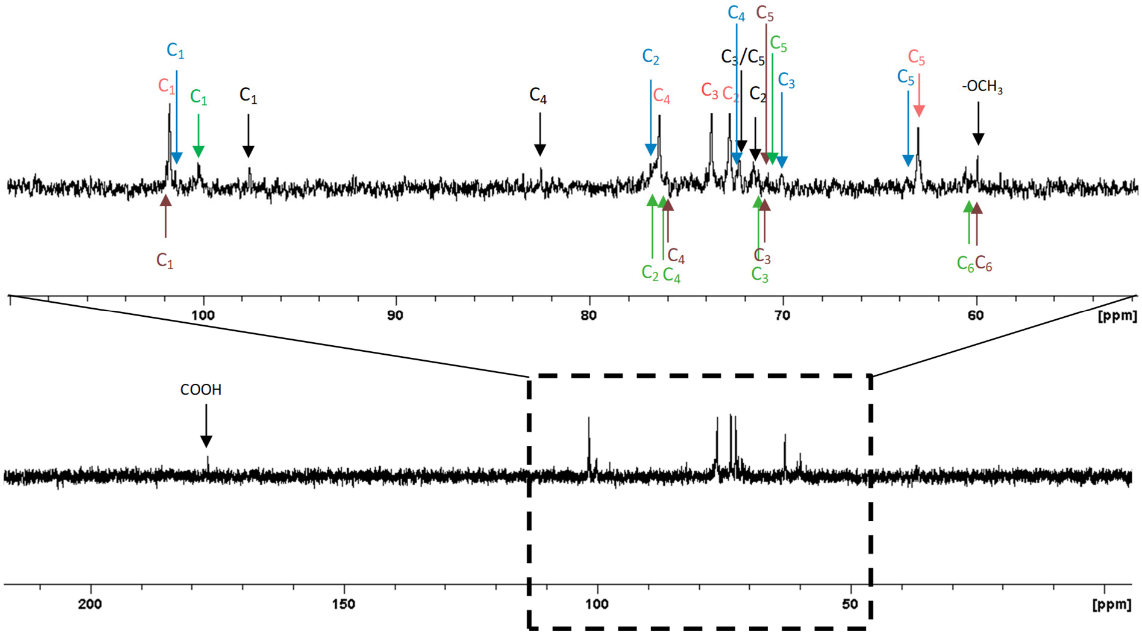

Figure S2. ^{13}C NMR spectrum of hemicelluloses extracted from the sulphite pulp in D_2O. (glucose units in brown, mannose units in green, non-substituted xylose units in red, glucuronic acid substituted xylose units in blue, glucuronic acid units in black. The number refers to the C-atom.

Table S2. Total signals assignation of hemicelluloses extracted from the sulfite pulp in D_2O.

Hemicelluloses	Monosaccharide units	Position	δ ^1H / ^{13}C (ppm)
Methylglucuronoxylans	β-Xylose non substitued	1	4.50 / 101.74 ($^3J_{H1-H2}$ = 7.88Hz)
		2	3.30 / 72.77
		3	3.57 / 73.74
		4	3.81 / 76.42
		5_{ax}	3.39 / 63.03
		$5_{éq}$	4.14 / 63.03
	β-Xylose substitued	1	4.64 / 101.45 ($^3J_{H1-H2}$ = 7.64Hz)
		2	3.43 / 76.46
		3	3.62 / 69.28
		4	3.62 / 72.42
		5_{ax}	3.30 / 65.12
		$5_{éq}$	3.98 / 65.12
	4-*O*-Methyl-α-Glucuronic acid	1	5.3 / 97.59 ($^3J_{H1-H2}$ = 3.6 Hz)
		2	3.58 / 71.30
		3	3.76 / 72.30
		4	3.23 / 82.55
		5	4.34 / 72.26
		-COOH	- / 176.80
		-OCH3	3.48 / 59.98
Glucomannans	β-Mannose	1	4.76 / 100.20 ($^3J_{H1-H2}$ = - Hz)
		2	3.56 / 76.36
		3	3.78 / 71.57
		4	3.57 / 74.95
		5	4.12 / 70.07
		6	3.72 et 3.94 / 60.58
	β-Glucose	1	4.60 / 101.90 ($^3J_{H1-H2}$ = 8 Hz)
		2	3.26 / 73.54
		3	3.78 / 71.57
		4	3.43 / 75.28
		5	4.12 / 70.07
		6	3.72 et 3.94 / 60.58

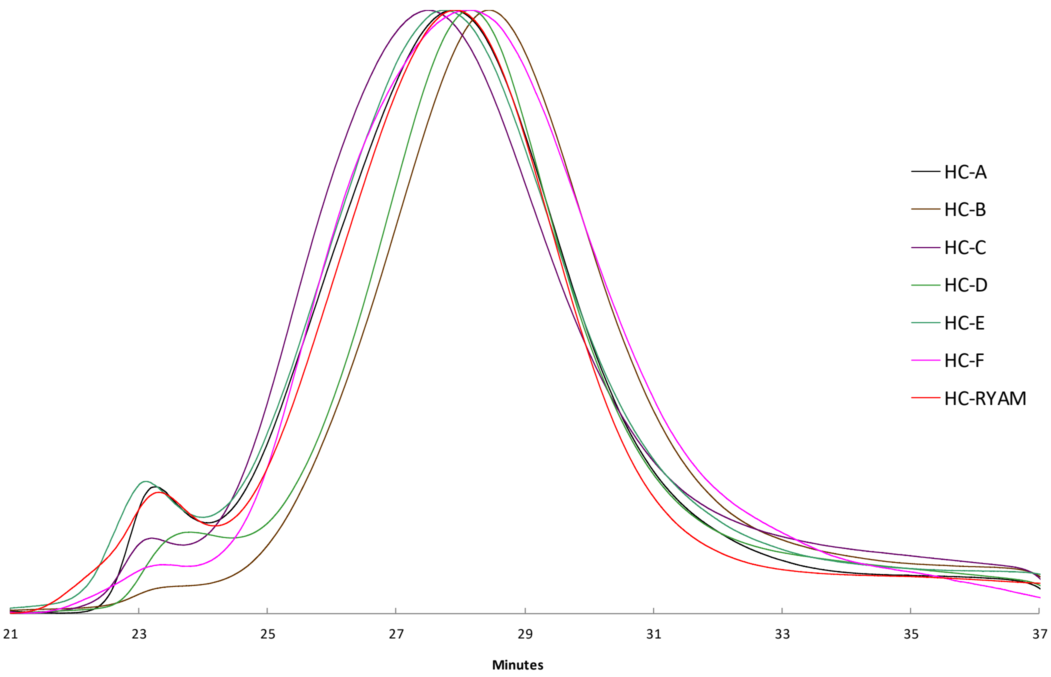

Figure S3. SEC-RI chromatograms of hemicelluloses extracted from the sulfite pulp.
